# Adaptive mediolateral control during split‐belt walking: Energetics of interlimb coordination and enhanced savings following acute intermittent hypoxia

**DOI:** 10.1113/EP093291

**Published:** 2026-03-21

**Authors:** Norah M. Nyangau, Alysha T. Bogard, Aviva K. Pollet, Andrew Q. Tan

**Affiliations:** ^1^ NeuroRecovery Lab University of Colorado Boulder Colorado USA; ^2^ Center for Neuroscience University of Colorado Boulder Colorado USA

**Keywords:** intermittent hypoxia, mediolateral stability, motor learning, split‐belt adaptation, walking energetics

## Abstract

Control of frontal plane mechanics requires active integration of sensory feedback to regulate stability in response to gait perturbations, such as split‐belt walking (SBW). In comparison to sagittal plane mechanics, mediolateral (ML) kinematic and kinetic adaptations to split‐belt perturbations are less extensively reported. Moreover, the associated metabolic cost of ML adaptations and the retention of previously learned adaptations, defined as motor savings, have not been examined concurrently. We investigated bilateral adaptations in step width and peak ML ground reaction forces to an initial SBW and metabolic cost. We also examined the retention of these adaptations during a subsequent SBW (adapt 2). Given evidence that priming the nervous system with acute intermittent hypoxia (AIH) enhances motor adaptation, we compared the magnitude of these adaptations after AIH. Legs on the fast and slow belt increased step width during initial SBW, but the magnitude of width reduced during adapt 2. Distinct kinetic modulation patterns emerged between legs as the initial increase in ML ground reaction forces was attenuated for the slow leg during the braking impulse phase and for the fast leg during the propulsive impulse phase. Metabolic cost reductions were positively associated with adaptations in ML force but not step width. During adapt 2, individuals who received AIH demonstrated greater reductions in step width and ML ground reaction forces during propulsion, suggesting enhanced motor savings. These asymmetrical ML kinetic adaptations contribute to stability and reduced metabolic cost during SBW. These insights might inform the design of training approaches to improve stability in clinical populations.

## Abstract (highlights)

- **What is the central question of this study?**
  How do mediolateral control strategies adapt during an initial and subsequent exposure to split‐belt walking (SBW)? Do mediolateral adaptations correspond to changes in metabolic cost? Does acute intermittent hypoxia (AIH) enhance the retention of these adaptations?

- **What is the main finding and its importance?**
  During SBW adaptation, step width initially increased, then narrowed towards baseline. Peak mediolateral forces were asymmetrically modulated and positively associated with reductions in metabolic cost. The retention of these adaptations was enhanced upon re‐exposure to SBW following AIH. These distinct kinematic and kinetic coordination strategies contribute to stability and energetics. Furthermore, AIH might facilitate motor adaptation, demonstrating its potential integration into motor rehabilitation.

## 1 INTRODUCTION

Maintenance of stability during walking is crucial for preventing falls in older adults (McIlroy & Maki, 1996; Rogers et al., 2001) and individuals with neurological impairments, such as spinal cord injury (Arora et al., 2019; Jørgensen et al., 2016). Improving frontal plane stability is especially crucial for populations with diminished motor control and adaptive capabilities (Arora et al., 2020; Fettrow et al., 2021; Sato & Choi, 2022). In contrast to the predominantly passive control of sagittal plane mechanics during walking (Kuo, 1999; Kuo & Donelan, 2010; McGeer, 1990), maintaining mediolateral (ML) stability in the frontal plane requires active, sensory feedback‐driven control of balance (Bauby & Kuo, 2000; Donelan et al., 2004; Kuo & Donelan, 2010). To enhance ML stability, able‐bodied individuals often adopt wider foot placement to increase their base of support (Bruijn & Van Dieën, 2018; Hak et al., 2013; McAndrew Young & Dingwell, 2012; Rawal & Singer, 2021). Indeed, individuals with neurological impairments who demonstrate reduced stability often adopt wider step widths in comparison to able‐bodied individuals (Curtze et al., 2024).

One approach to assess ML stability is through split‐belt walking (SBW), which uses single‐belt speed perturbations to induce transient walking asymmetries (Buurke et al., 2018; Cornwell et al., 2024; Fettrow et al., 2021). These perturbations induce error‐based motor learning (Leech et al., 2022; Malone et al., 2012; Reisman et al., 2005; Roemmich & Bastian, 2015), as evidenced by adaptations in step length asymmetry and step time asymmetry (Bogard et al., 2023; Finley et al., 2013; Malone et al., 2012; Sánchez et al., 2021). Several factors can influence the capacity for adaptation, including motor impairment (Malone & Bastian, 2014; Sánchez & Finley, 2018), sensory feedback (Gonzalez‐Rubio et al., 2019; Shin & Chung, 2022) and neuroplastic changes induced by brief exposures to low‐oxygen air, known as acute intermittent hypoxia (AIH) (Bogard et al., 2023, 2025). Although step asymmetry and anterior–posterior (AP) ground reaction forces (GRF) have been studied extensively during destabilizing walking tasks (Ogawa et al., 2015; Yokoyama et al., 2018), corresponding adaptations in step widths and ML GRFs, key contributors to ML stability (Rawal & Singer, 2021), have received comparatively less attention.

Additionally, frontal plane adaptations during SBW have been documented only during a single SBW exposure (Brinkerhoff et al., 2024; Buurke et al., 2018, 2019b; Cornwell et al., 2024; Darter et al., 2018). In the sagittal plane, able‐bodied individuals demonstrate faster adaptation during subsequent exposure to the same perturbation within minutes after the initial exposure, known as motor savings (Bogard et al., 2023; Leech et al., 2018; Malone et al., 2011; Roemmich & Bastian, 2015). However, motor savings of ML stability measures, such as step widths and ML GRF, have not been examined directly. The first objective of this study was to examine changes in ML coordination strategies across an initial adaptation (adapt 1) and subsequent adaptation (adapt 2) to SBW to characterize motor adaption and motor savings, respectively.

Another important feature of SBW adaptation is changes in net metabolic power (Butterfield & Collins, 2022; Sánchez et al., 2017; Stenum & Choi, 2020). During SBW, we and others have demonstrated experimentally and with biomechanical simulation that net metabolic power decreases as participants adapt interlimb coordination strategies in the sagittal plane (Bogard et al., 2023; Butterfield & Collins, 2022; Finley et al., 2013; Price et al., 2023; Sánchez et al., 2019). Furthermore, the net metabolic cost of adaptation and savings is reduced following AIH (Bogard et al., 2023; 2025). However, sagittal plane mechanics do not account fully for the variability in net metabolic power (Bogard & Tan, 2024). Frontal plane adaptations can be energetically costly (Donelan et al., 2001), and external ML stabilization has been estimated to reduce net metabolic power during normal walking (Dean et al., 2007; Donelan et al., 2004). Although individuals might exploit passive ML dynamics to minimize energetic effort during adaptation (Fettrow et al., 2021), changes in ML margin of stability or step width during SBW do not predict net metabolic power consistently (Buurke et al., 2018). Thus, the second objective of this study was concurrently to characterize the corresponding energetic cost of the ML adaptations during the initial motor adaptation phase and motor savings phase.

We demonstrated recently that AIH enhances sagittal plane adaptation and elicits greater reductions in net metabolic power during SBW (Bogard et al., 2023). AIH is a promising intervention that promotes recovery of walking in humans with incomplete spinal cord injury (Tan et al., 2021; Tan, Barth et al., 2020). One plausible mechanism underlying such gains in motor function is the facilitation of descending neural excitability that might influence motor adaptation as observed in able‐bodied individuals (Bogard et al., 2024; 2025). Notably, enhanced corticospinal plasticity has been linked to increased motor output (Bogard et al., 2024, 2025). However, it is unclear whether AIH‐induced plastic changes that facilitate greater retention of adaptive motor strategies can promote similar enhancements in ML control. Therefore, the final objective of this study was to investigate whether AIH exposure enhances frontal plane motor adaptations and savings and whether such changes drive greater reductions in net metabolic power.

To examine neuromechanical strategies that contribute to both stability and metabolic cost, we measured adaptations in step width, peak ML GRF and net metabolic power during an initial and subsequent exposure to an SBW perturbation to characterize motor adaptation and motor savings. We hypothesized that participants would concurrently reduce the initial ML adjustments along with net metabolic power and demonstrate motor savings of these reductions upon subsequent exposure. Given that AIH has been shown to improve sagittal plane adaptation with improved metabolic efficiency (Bogard et al., 2023), we further hypothesized that the reductions in step width, peak ML GRF and net metabolic power would be more prominent in the AIH group relative to the Control group. These findings might reveal insights into adaptive ML control when stability is challenged and provide further insight into the potential use of AIH as a priming intervention to improve walking stability and reduce fall risk in clinical populations.

## 2 MATERIALS AND METHODS

### 2.1 Ethical approval

Thirty able‐bodied individuals with no prior history of neurological impairments were recruited for the study (Table 1). All participants provided signed informed consent approved by the Colorado Multiple Institutional Review Board (COMIRB no. 20‐0689). The procedures complied with the standards of the *Declaration of Helsinki*, and the study was registered on clinicaltrials.org (NCT05341466).

**TABLE 1:** Participant demographics.

Participant	Group	Age (years)	Sex	Height (cm)	Weight (kg)
1	Control	21	F	161.29	60.33
2	Control	35	M	185.42	84.82
3	Control	22	M	185.42	79.37
4	Control	25	F	162.56	61.23
5	Control	19	M	182.88	72.57
6	Control	33	F	172.72	65.32
7	Control	28	M	185.42	108.86
8	Control	30	F	162.56	51.25
9	Control	25	F	175.26	79.38
10	Control	30	M	177.80	72.57
11	Control	20	M	182.88	83.91
12	Control	30	M	185.42	92.99
13	Control	21	F	165.10	62.14
14	Control	21	M	185.42	77.11
15	Control	20	F	162.56	56.70
16	AIH	27	M	170.18	86.18
17	AIH	24	M	180.00	73.00
18	AIH	21	F	154.94	63.50
19	AIH	26	F	162.56	52.16
20	AIH	23	F	154.94	49.89
21	AIH	24	F	162.56	54.88
22	AIH	27	M	190.50	75.00
23	AIH	21	F	167.64	58.97
24	AIH	21	M	185.42	76.20
25	AIH	20	F	162.56	52.16
26	AIH	26	F	165.10	70.31
27	AIH	22	M	185.42	82.10
28	AIH	25	M	180.34	72.57
29	AIH	24	F	167.64	64.86
30	AIH	22	F	167.64	54.43

*Note*: Thirty able‐bodied individuals were separated into either an AIH group (*n* = 15, 9 females, 6 males, mean± SD age = 23.5 ± 3 years, body mass = 65.3 ± 11.8 kg, height = 170 ± 11.2 cm) or a Control group (*n* = 15, 7 females, 8 males, age 25.3 ± 5.3 years, body mass = 73.5 ± 14.9 kg, height 175 ± 10.1 cm).

Abbreviations: AIH, acute intermittent hypoxia; F, female; M, male.

### 2.2 Participants

A priori power analyses were conducted using pilot data from the present study to determine the required sample size for detecting both between‐ and within‐group differences across two adaptation phases (G*Power v.3.1.9.7, Düsseldorf, Germany) (Erdfelder et al., 2009). For step width, results indicated that a minimum of 13 participants per group would be adequate to observe significant effects [power = 0.85, Cohen's *f* = 0.31, α = 0.05, *F*(1,11) = 4.26]. For ML GRF, a sample size of 12 participants per group was determined to be sufficient [power = 0.85, Cohen's *f* = 0.33, α = 0.05, *F*(1,10) = 4.30]. Participants were randomized into either a Control group (*n* = 15, 7 females, 8 males, mean ± SD age 25.3 ± 5.3 years, body mass = 73.5 ± 14.9 kg, height 175 ± 10.1 cm) or an AIH group (*n* = 15, 9 females, 6 males, age = 23.5 ± 2.3 years, body mass = 65.3 ± 11.8 kg, height 170 ± 11.2 cm). We excluded individuals who had prior experience with SBW to avoid retained adaptation strategies (Buurke et al., 2022; Roemmich & Bastian, 2015). Additional criteria for exclusion included altitude sensitivity, cardiovascular or pulmonary diseases, syncope, or being pregnant at the time of the study.

### 2.3 Protocols

#### 2.3.1 Gait mechanics

Kinematic data were recorded with a 10‐camera system at a rate of 100 Hz (Vicon Nexus v.2.8.1; Vicon Motion Systems, Denver, CO, USA). Time‐synchronized kinetic variables were measured on an instrumented split‐belt treadmill at a sampling rate of 1000 Hz, and the belt speeds were controlled independently (M‐Gait, D‐flow v.3.34.3; Motek Medical, Houten, The Netherlands). Thirty‐four reflective markers were placed on shank and thigh grid clusters and on the following anatomical landmarks: anterior and posterior superior iliac spines, iliac crests, greater trochanters, medial and lateral femoral epicondyles, medial and lateral malleoli, calcanei and first and fifth metatarsals (Montgomery & Grabowski, 2018). A mirror was positioned in front of the treadmill to help individuals avoid crossing their feet onto the opposite belt. Participants were instructed to avoid using the handrails unless necessary for safety (Buurke et al., 2019a) and were secured with a single passive harness that neither affected movement nor provided body weight support.

#### 2.3.2 Split‐belt walking protocol

**FIGURE 1:**
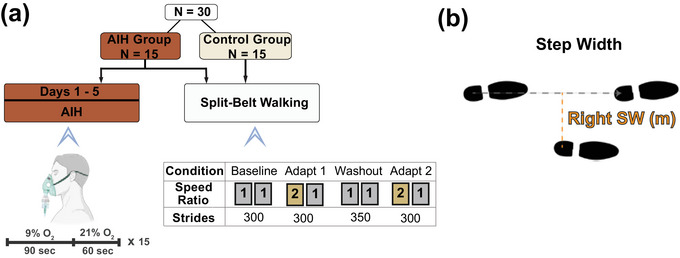
Protocol figure. (a) Individuals in the AIH intervention group (*n* = 15) were administered 90 s bouts of 9% oxygen alternated with 60 s of room air for 15 total cycles, repeated for 5 days consecutively. All participants (*n* = 30) then performed the split‐belt walking protocol for the following conditions: ‘baseline’ at tied‐belt, ‘adapt 1’ at a 2:1 speed ratio, ‘washout’ at tied‐belt, and ‘adapt 2’ at a 2:1 speed ratio. (b) Schematic diagram for calculating right step width. Step widths were calculated as the perpendicular distance between a stride vector formed by the positions of the calcaneus marker from consecutive heel‐strikes of the contralateral limb and the calcaneus marker at heel‐strike of the ipsilateral limb. Abbreviations: AIH, acute intermittent hypoxia; SW, step width.

All participants completed a single SBW protocol as described by Bogard et al. (2023), which included two tied‐belt and two split‐belt walking conditions for a total of four trials (Figure 1a). A single belt was randomly selected to be sped up without warning and remained consistent across the two split‐belt walking trials (Reisman et al., 2005). To minimize potential bias, leg dominance was not considered, but we instead randomized which leg was assigned to the fast belt and ensured that subjects remained blinded to the leg assignment. The first trial was ‘baseline’, which included walking at a tied‐belt speed of 1.0 m/s for 300 strides. Following baseline, participants performed an ‘adapt 1’ trial that involved tied‐belt walking at 1.0 m/s for 15–30 strides before a sudden increase of one belt to 2.0 m/s for 300 strides of SBW at a 2:1 belt speed ratio. Next, the participants performed a ‘washout’ trial, in which they walked with tied‐belt speed of 1.0 m/s for 350 strides. Finally, participants performed an ‘adapt 2’ trial by walking at 1.0 m/s tied‐belt speed for 15–30 strides followed by a second exposure to the same split‐belt perturbation at a 2:1 belt speed ratio for 300 strides. The trials were performed consecutively, stopping after each trial with an optional 1 min break, during which individuals were instructed to stand still (Leech et al., 2018; Sombric & Torres‐Oviedo, 2020).

#### 2.3.3 Acute intermittent hypoxia

The AIH group received 5 consecutive days of low‐oxygen treatment followed by SBW 15 min after the final AIH exposure (Figure 1a). For the treatment, low‐oxygen air was delivered through an altitude generator (HYP 123; Hypoxico, Inc., USA), while trained research staff continuously monitored heart rate (HR) and oxygen saturation levels. Blood pressure (BP) was taken every five normoxic cycles (Root Monitor; Masimo; Irvine, CA, USA). Similar to the study by Tan et al. (2021), AIH consisted of 90 s bouts of hypoxic air (9% O_2_) followed by 60 s bouts of normoxic air (21% O_2_) for a total of 15 cycles (Figure 1a). AIH treatment was paused if individuals desaturated below an oxygen saturation level of 70% and resumed when they resaturated above 80% (Tan, Papadopoulos et al., 2020). The experiment was discontinued if any of the following conditions occurred: systolic BP exceeded 140 mmHg, diastolic BP exceeded 90 mmHg, or HR surpassed 160 beats/min (Tan, Papadopoulos et al., 2020). Termination criteria also included participant‐reported or visible signs of adverse events such as dizziness, numbness, tinnitus, blurred vision or diaphoresis. The AIH protocol was repeated for 5 consecutive days, on which participants came in within 30 min of the same time each day. All participants tolerated the hypoxic dose and completed the full procedure.

### 2.4 Data processing and analysis

Metabolic rate was measured using an open‐circuit spirometry system (TrueOne 2400; ParvoMedics Inc., Salt Lake City, UT, USA). The rates of oxygen consumption (${\dot{V}}_{O_{2}}$; in litres per second) and carbon dioxide production (${\dot{V}}_{{\mathrm{CO}}_{2}};\;\mathrm{in}\;\mathrm{litres}\;\mathrm{per}\;\mathrm{second}$) were used to calculate respiratory exchange ratios (RER =$\;{\dot{V}}_{{\mathrm{CO}}_{2}}\;/{\dot{V}}_{O_{2}}$). All participants maintained an RER below one during SBW, indicating a primary contribution of aerobic metabolic pathways (Huang et al., 2012; Ramos‐Jiménez et al., 2008). Resting metabolic rate was estimated from the average ${\dot{V}}_{O_{2}}$ and ${\dot{V}}_{{\mathrm{CO}}_{2}}$ of each individual during the last 2 min of a 5 min standing trial for the AIH (1.51 ± 0.26 W/kg) and control (1.50 ± 0.20 W/kg) groups and subtracted from the metabolic data for each trial. Given that steady‐state conditions were confirmed from RER values, we estimated the energetic cost during walking trials by calculating metabolic power using the regression formula provided in Equation 1 (Péronnet & Massicotte, 1991).

(1) $$\mathrm{Metabolic}\;\mathrm{power}\;\left(W\right)=16.98\;{\dot{V}}_{O_{2}}+6.98\;{\dot{V}}_{{\mathrm{CO}}_{2}}$$

Net metabolic power (in watts per kilogram) was obtained from the difference between metabolic power and average resting metabolic rate and normalized to body mass (Finley et al., 2013). Similar to prior work, the time needed for net metabolic power to stabilize to its average steady‐state value was validated for each individual to be 1 min, hence the initial 60 s of each trial were excluded (Finley et al., 2013). This transient period reflects the duration of expired gas travelling through a 2.38 L expiratory tube to reach a 4 L chamber, which corresponds to a delay of ∼16 s, assuming an average ventilatory rate of 25 L/min.

Processing of motion‐capture markers included manual labelling and interpolation of position trajectories using the pattern fill filter embedded in Vicon Nexus software (Camargo et al., 2020). Lower‐body gait models were created using the pelvis and bilateral lower extremity anatomical segments and analysed using custom pipelines created in Visual3D (v.2021.11.3; HAS‐motion Inc., Germantown, MD, USA). The GRF were low‐pass filtered using a fourth‐order zero‐lag Butterworth filter with a cut‐off frequency of 20 Hz, applied bidirectionally (Gottschall & Kram, 2003; Zeni et al., 2008). In Visual3D, we identified heel‐strike and push‐off gait events at vertical GRF thresholds of 30 N to reduce false event detections in trials with higher baseline noise (Karakasis & Artemiadis, 2021). Midstance events were defined at the zero‐crossing of anterior–posterior GRF for each stride, marking the transition from braking into the propulsive phase of the gait (Masani et al., 2002). Therefore, each stride contained intervals between heel‐strike and midstance defined as the braking phases, and between midstance and push‐off as the propulsive phases. We obtained peak ML GRF values during both the braking and propulsive phases at each stride. Step widths were calculated with the Visual3D system algorithm as the perpendicular distance between a stride vector formed by the positions of the calcaneus marker from consecutive heel‐strikes of the contralateral limb and the calcaneus marker at heel‐strike of the ipsilateral limb (Figure 1b) (Rawal & Singer, 2021; Reimold et al., 2020).

**FIGURE 2:**
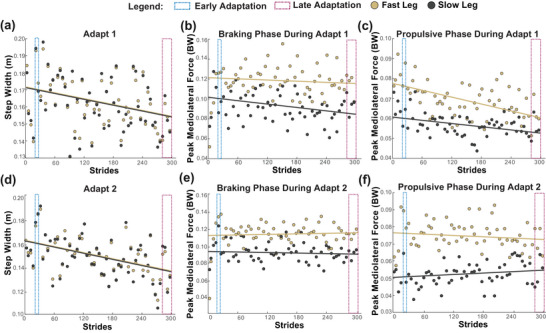
Data from a representative subject. A subject's (*n* = 1) full adaptation of step width (in metres) (a, d) and peak ML GRF (in units of body weight) during the braking (b, e) and propulsive (c, f) phases of gait during adapt 1 and adapt 2. Abbreviations: AIH, acute intermittent hypoxia; BW, body weight; GRF, ground reaction force; ML, mediolateral.

Averaged values of step width and peak ML GRF were calculated at different adaptation phases of each trial. We analysed the last 20 strides of baseline to compare with the SBW trials. Adapt 1 and adapt 2 SBW trials were divided into an ‘early’ adaptation phase consisting of the first five strides immediately following the change in belt speed, and a ‘late’ adaptation phase consisting of the last 20 strides (Finley et al., 2013; Leech et al., 2018). We defined the progression of motor adaptation from early to late phase within adapt 1 as motor learning. To characterize the short‐term retention of motor strategies, we compared the early and late adaptation phases across both SBW trials defined as motor savings (Donelan et al., 2002). ‘Early savings’ compared motor adaptions between early adapt 1 and early adapt 2, and ‘late savings’ compared motor adaptations between late adapt 1 and late adapt 2. Figure 2 (a‐f) illustrates full adaptation for a representative subject as they adjust step width and peak ML GRF metrics during adapt 1 and adapt 2 trials.

### 2.5 Statistical analysis

Kinematic, kinetic and metabolic data were processed in MATLAB (R2023b; The MathWorks, Inc., Natick, MA, USA). Summary statistics are provided in Table 2. Statistical analysis was performed in R Studio (v.2024.04.2), with the significance set to *P <* 0.05. Normality and homogeneity of the data were tested using Shapiro–Wilks and Levene's tests, respectively. Linear mixed models (LMMs) were used to evaluate changes in biomechanical and metabolic adaptations. Separate LMMs were fitted for step width and peak ML GRF during the braking and propulsive phases of gait. The models included leg speed (fast vs. slow) and adaptation phase (early vs. late) as the fixed effects and a random intercept assigned to each subject to account for within‐subject variability. The models were run separately for adapt 1 and adapt 2. For main effects and interactions that reached statistical significance (*P <* 0.05), *post hoc* analyses were conducted using Tukey's honest significant difference (HSD) method that applied corrections to adjust for multiple comparisons, mitigating the risk of type I errors. To assess whether participants returned to baseline walking during de‐adaptation, Student's paired *t*‐tests were conducted between baseline and washout conditions for step width and peak ML GRF metrics.

**TABLE 2:** Summary statistics.

			Adapt 1		Adapt 2	
			Early	Late	Early	Late
Step width, m		Fast leg	0.194 (0.0350)	0.161 (0.0222)	0.172 (0.0235)	0.163 (0.0193)
			−0.005 (−0.032, 0.021)	0.018 (0.002, 0.033)	0.012 (−0.005, 0.030)	0.009 (−0.005, 0.024)
		Slow leg	0.193 (0.0367)	0.161 (0.0220)	0.174 (0.0241)	0.163 (0.0189)
			0.002 (−0.026, 0.030)	0.017 (0.002, 0.033)	0.011 (−0.007, 0.029)	0.009 (−0.005, 0.023)
Peak ML GRF, BW	Braking phase	Fast leg	0.135 (0.035)	0.135 (0.026)	0.129 (0.026)	0.134 (0.021)
			−0.014 (−0.040, 0.012)	0.002 (−0.018, 0.022)	−0.007 (−0.026, 0.013)	−0.001 (−0.017, 0.015)
		Slow leg	0.130 (0.040)	0.092 (0.016)	0.099 (0.019)	0.092 (0.016)
			−0.022 (−0.051, 0.007)	−0.005 (−0.017, 0.006)	−0.007 (−0.021, 0.008)	−0.005 (−0.017, 0.007)
	Propulsive phase	Fast leg	0.123 (0.034)	0.072 (0.019)	0.090 (0.017)	0.072 (0.012)
			−0.010 (−0.035, 0.015)	0.007 (−0.007, 0.021)	0.0004 (−0.013, 0.014)	0.002 (−0.008, 0.011)
		Slow leg	0.078 (0.027)	0.070 (0.015)	0.066 (0.013)	0.070 (0.014)
			−0.010 (−0.030, 0.010)	0.001 (−0.010, 0.012)	0.0004 (−0.009, 0.010)	0.0005 (−0.010, 0.011)

*Note*: Mean (SD) for all participants (*n* = 15 AIH; *n* = 15 Control) and mean difference (CI) between groups (Control ‐ AIH) with 95% confidence intervals of step width (in meters) and peak mediolateral ground reaction force (body weight) for the fast and slow legs during early and late phases of adapt 1 and adapt 2. Abbreviations: AIH, acute intermittent hypoxia; BW, body weight; GRF, ground reaction force; ML, mediolateral.

To investigate the relationships between biomechanical adaptations and net metabolic power, linear regression analyses were used to compare changes in net metabolic power with corresponding changes in step width and peak ML GRF during the braking and propulsive phases during motor adaptation and motor savings, separately for the fast and slow legs.

#### 2.5.1 Between‐group statistical analyses

To investigate the effects of AIH intervention on motor savings, additional LMMs incorporated fixed effects of intervention group (AIH vs. Control) and motor savings phases (early adapt 1 vs. early adapt 2; late adapt 1 vs. late adapt 2) on step width and peak ML GRF during the braking and propulsive phases of gait, independently for the fast leg and slow leg. Tukey's HSD *post hoc* analyses were performed separately for the fast and slow legs within the AIH and Control groups to identify significant pairwise differences. To assess the magnitude of metabolic savings further, Welch's *t*‐tests were conducted between groups during early and late savings.

Likewise, linear regression analyses were performed within the AIH group to assess how changes in biomechanical variables corresponded to changes in net metabolic power. For each leg (fast and slow) and for both phases (early and late adaptation), we compared changes in step width and peak ML GRF during the braking and propulsive phases to reductions in net metabolic power.

## 3 RESULTS

### 3.1 Kinematic adaptations: Step width

**FIGURE 3:**
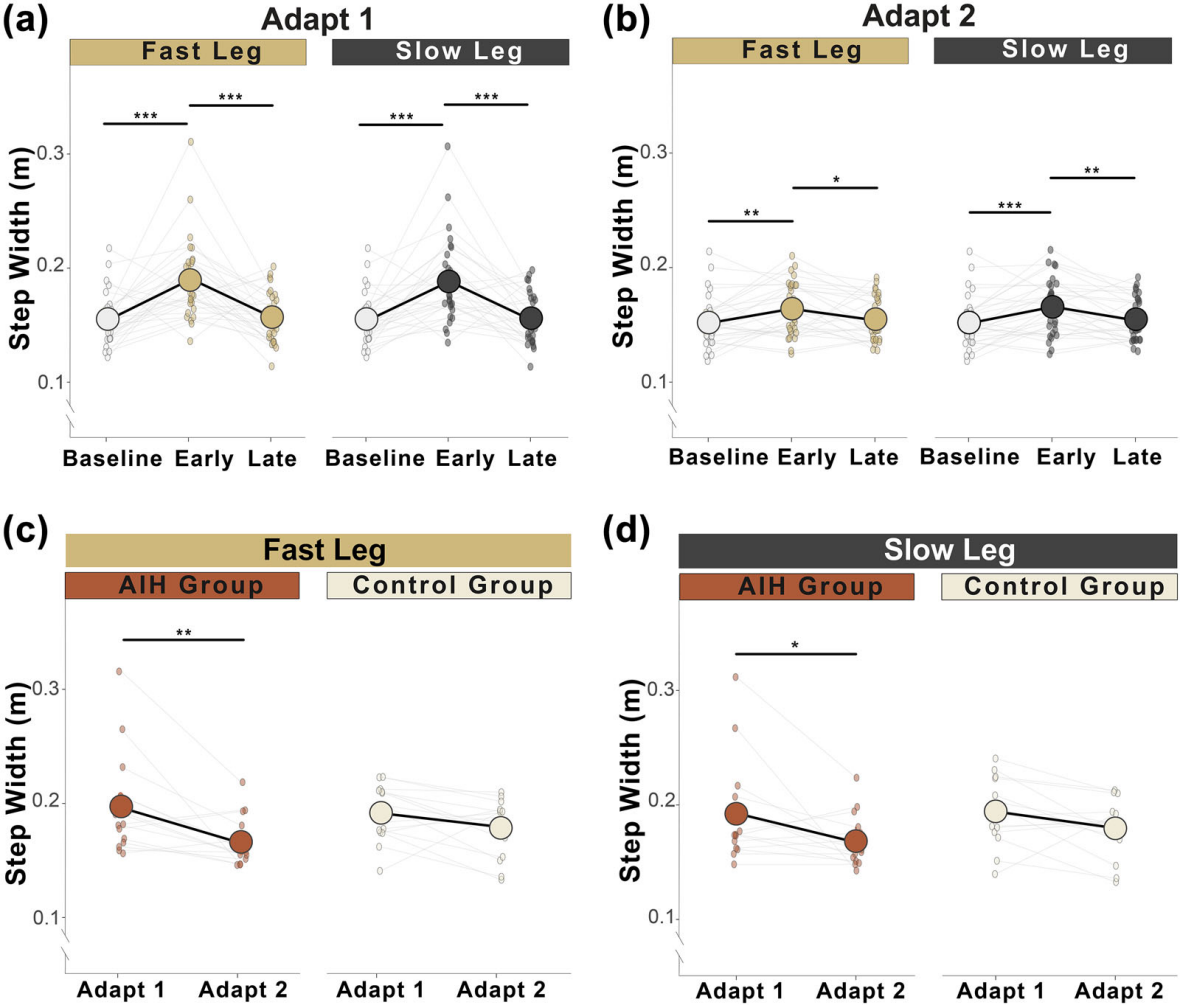
Step width adaptation. All participants (*n* = 30) increased their step width (in metres) from baseline during early adaptation, then significantly narrowed during late adaptation of adapt 1 (a) and likewise in adapt 2 (b) for both legs. The AIH group demonstrated early savings of step width in both the fast leg (c) and the slow leg (d) compared with control subjects. Circles represent mean step width, and dots show the average step width for each individual. *P < 0.05, **P < 0.01 and ***P < 0.001. Abbreviations: AIH, acute intermittent hypoxia; BW, body weight; GRF, ground reaction force; ML, mediolateral.

During adapt 1 and adapt 2, there was a main effect of adaptation phase on step width, but neither leg speed nor an interaction of leg speed and phase had significant effects (Table 3). *Post hoc* comparisons revealed that both the fast and slow legs significantly increased width from baseline to early adaptation, then decreased during late adaptation in both adapt 1 (Figure 3a) and adapt 2 (Figure 3b; Table 4).

**TABLE 3:** Linear mixed model analyses.

(a)			Motor adaptation LMM		
			Leg speed	Phase	Leg speed × phase
Step width, m		Adapt 1	*F*(1,145) = 0.027, *P =* 0.871	*F*(2,145) = 44.2, *P = 1.00 × 10^−15 ***^	*F*(2,145) = 0.013, *P =* 0.987
		Adapt 2	*F*(1,145) = 0.063, *P =* 0.802	*F*(2,145) = 14.7, *P =* 1.51 × 10^−6 ***^	*F*(2,145) = 0.067, *P =* 0.935
Peak ML GRF, BW	Braking phase	Adapt 1	*F*(1,145) = 34.1, *P =* 3.31 × 10^−8 ***^	*F*(2,145) = 109, *P =* 1.20 × 10^−29 ***^	*F*(2,145) = 24.0, *P =* 1.02 × 10^−9 ***^
		Adapt 2	*F*(1,145) = 186, *P =* 9.18 × 10^−28 ***^	*F*(2,145) = 133, *P =* 1.42 × 10^−33 ***^	*F*(2,145) = 50.6, *P =* 2.15 × 10^−17 ***^
	Propulsive phase	Adapt 1	*F*(1,145) = 38.8, *P =* 4.78 × 10^−9 ***^	*F*(2,145) = 54.3, *P =* 2.49 × 10^−18 ***^	*F*(2,145) = 33.2, *P =* 1.34 × 10^−12 ***^
		Adapt 2	*F*(1,145) = 40.7, *P =* 2.24 × 10^−9 ***^	*F*(2,145) = 9.32, *P =* 1.56 × 10^−4 ***^	*F*(2,145) = 31.2, *P =* 5.45 × 10^−12 ***^
(b)			Early savings LMM		
			Group	Phase	Group × phase
Step width, m		Fast leg	*F*(1,28) = 0.134, *P =* 0.717	***F*(1,28) = 13.4,** *P =* 0.00102 **	*F*(1,28) = 2.20, *P =* 0.149
		Slow leg	*F*(1,28) = 0.484, *P =* 0.492	***F*(1,28) = 9.27,** *P =* 0.00503 **	*F*(1,28) = 0.510, *P =* 0.481
Peak ML GRF, BW	Braking phase	Fast leg	*F*(1,28) = 0.975, *P =* 0.332	*F*(1,28) = 2.03, *P =* 0.165	*F*(1,28) = 0.647, *P =* 0.428
		Slow leg	*F*(1,28) = 2.04, *P =* 0.164	*F*(1,28) = 42.1, *P =* 4.95 × 10^−7 ***^	*F*(1,28) = 2.68, *P =* 0.113
	Propulsive phase	Fast leg	*F*(1,28) = 0.349, *P =* 0.559	*F*(1,28) = 43.0, *P =* 4.15 × 10^−7 ***^	*F*(1,28) = 1.10, *P =* 0.303
		Slow leg	*F*(1,28) = 0.631, *P =* 0.434	*F*(1,28) = 7.08, *P =* 0.0128 *	*F*(1,28) = 1.54, *P =* 0.225

*Note*: (a) Main effects of leg speed and adaptation phase on step width (in metres) and peak mediolateral ground reaction force (units of body weight) during adapt 1 and adapt 2 for all participants (*n* = 30); and (b) main effects of group and phase for the fast and slow legs during early savings (early adapt 1 vs. early adapt 2) for the AIH (*n* = 15) and Control (*n* = 15) groups. * *P* < 0.05, ** *P* < 0.01, *** *P* < 0.001.

Abbreviations: AIH, acute intermittent hypoxia; BW, body weight; GRF, ground reaction force; LMM, Linear mixed model; ML, mediolateral.

**TABLE 4:** Tukey's *post hoc* analyses.

(a)			Between‐phase pairwise comparisons					
			Adapt 1			Adapt 2		
			Baseline vs. early	Baseline vs. late	Early learning vs. late	Baseline vs. early	Baseline vs. late	Early learning vs. late
Step width, m		Fast leg	*t*(145) = −5.95, *P =* 5.87 × 10^−8 ***^	*t*(145) = −0.187, *P =* 0.981	*t*(145) = 5.76, *P =* 1.46 × 10^−7 ***^	*t*(145) = −3.44, *P =* 2.15 × 10^−3 **^	*t*(145) = −0.855, *P =* 0.669	*t*(145) = 2.59, *P *= 0.0284 *
		Slow leg	*t*(145) = −5.73, *P =* 1.70 × 10^−7 ***^	*t*(145) = −0.124, *P =* 0.991	*t*(145) = 5.60, *P =* 3.09 × 10^−7 ***^	*t*(145) = −3.89, *P =* 4.50 × 10^−4 ***^	*t*(145) = −0.847, *P =* 0.675	*t*(145) = 3.04, *P *= 0.00785 **
Peak ML GRF, BW	Braking phase	Fast leg	*t*(145) = −10.9, *P =* 2.09 × 10^−14 ***^	*t*(145) = −10.9, *P =* 2.30 × 10^−14 ***^	*t*(145) = 0.063, *P =* 0.998	*t*(145) = −15.06, *P =* 3.55 × 10^−15 ***^	*t*(145) = −16.73, *P =* 3.55 × 10^−15 ***^	*t*(145) = −1.67, *P =* 0.220
		Slow leg	*t*(145) = −9.79, *P =* 3.16 × 10^−14 ***^	*t*(145) = −1.87, *P =* 0.151	*t*(145) = 7.92, *P =* 1.72 × 10^−12 ***^	*t*(145) = −5.28, *P =* 1.36 × 10^−6 ***^	*t*(145) = −2.90, *P =* 0.0121 *	*t*(145) = 2.39, *P =* 0.0476 *
	Propulsive phase	Fast leg	*t*(145) = −11.23, *P =* 1.62 × 10^−14 ***^	*t*(145) = 0.245, *P =* 0.967	*t*(145) = 11.47, *P =* 1.21 × 10^−14 ***^	*t*(145) = −6.98, *P =* 2.92 × 10^−10 ***^	*t*(145) = 0.616, *P =* 0.812	*t*(145) = 7.60, *P =* 1.04 × 10^−11 ***^
		Slow leg	*t*(145) = −0.985, *P =* 0.588	*t*(145) = 0.792, *P =* 0.708	*t*(145) = 1.78, *P =* 0.181	*t*(145) = 3.13, *P =* 0.00601 **	*t*(145) = 1.56, *P =* 0.267	*t*(145) = −1.57, *P =* 0.262
(b)			Within leg speed pairwise comparisons					
			Adapt 1			Adapt 2		
			Baseline	Early	Late	Baseline	Early	Late
Step width, m			*t*(145) = 0.00, *P =* 1.00	*t*(145) = 0.220, *P =* 0.826	*t*(145) = 0.063, *P =* 0.950	*t*(145) = 0.00, *P =* 1.00	*t*(145) = −0.443, *P =* 0.658	*t*(145) = 0.008, *P =* 0.993
Peak ML GRF, BW	Braking phase		*t*(145) = 0.00, *P =* 1.00	*t*(145) = 1.13, *P =* 0.261	*t*(145) = 8.99, *P =* 1.26 × 10^−15 ***^	*t*(145) = 0.00, *P =* 1.00	*t*(145) = 9.78, *P =* 1.16 × 10^−17 ***^	*t*(145) = 13.8, *P =* 2.73 × 10^−28 ***^
	Propulsive phase		*t*(145) = 0.00, *P =* 1.00	*t*(145) = 10.2, *P =* 7.27 × 10^−19 ***^	*t*(145) = 0.547, *P =* 0.585	(145) = 0.00, *P =* 1.00	*t*(145) = 10.1, *P =* 1.64 × 10^−18 ***^	*t*(145) = 0.942, *P =* 0.348
(c)			Early savings pairwise comparisons					
			Fast leg		Slow leg			
			AIH group	Control group	AIH group	Control group		
Step width, m			*t*(28) = 3.64, *P =* 0.00109 **	*t*(28) = 1.55, *P =* 0.134	*t*(28) = 2.66, *P =* 0.0128 *	*t*(28) = 1.65, *P =* 0.111		
Peak ML GRF, BW	Braking phase		*t*(28) = 1.58, *P =* 0.126	*t*(28) = 0.438, *P =* 0.665	*t*(28) = 5.75, *P =* 3.63 × 10^−6 ***^	*t*(28) = 3.43, *P =* 0.00188 **		
	Propulsive phase		*t*(28) = 5.38, *P =* 9.88 × 10^−6 ***^	*t*(28) = 3.89, *P =* 5.57 × 10^−4 ***^	*t*(28) = 2.76, *P =* 0.0101 *	*t*(28) = 1.00, *P =* 0.324		

*Note*: Pairwise comparisons of step width (in metres) and peak ML GRF (in units of body weight) for the fast and slow legs: (a) between adaptation phases; (b) within leg speed (fast vs. slow) during adapt 1 and adapt 2 for all participants (*n* = 30); and (c) during early savings (early adapt 1 vs. early adapt 2) within the AIH (*n* = 15) and Control (*n* = 15) groups. * *P* < 0.05, ** *P* < 0.01, *** *P* < 0.001.

Abbreviations: AIH, acute intermittent hypoxia; BW, body weight; GRF, ground reaction force; ML, mediolateral.

### 3.2 Kinetic adaptations: Peak mediolateral force

#### 3.2.1 Motor adaptation during braking phase

**FIGURE 4:**
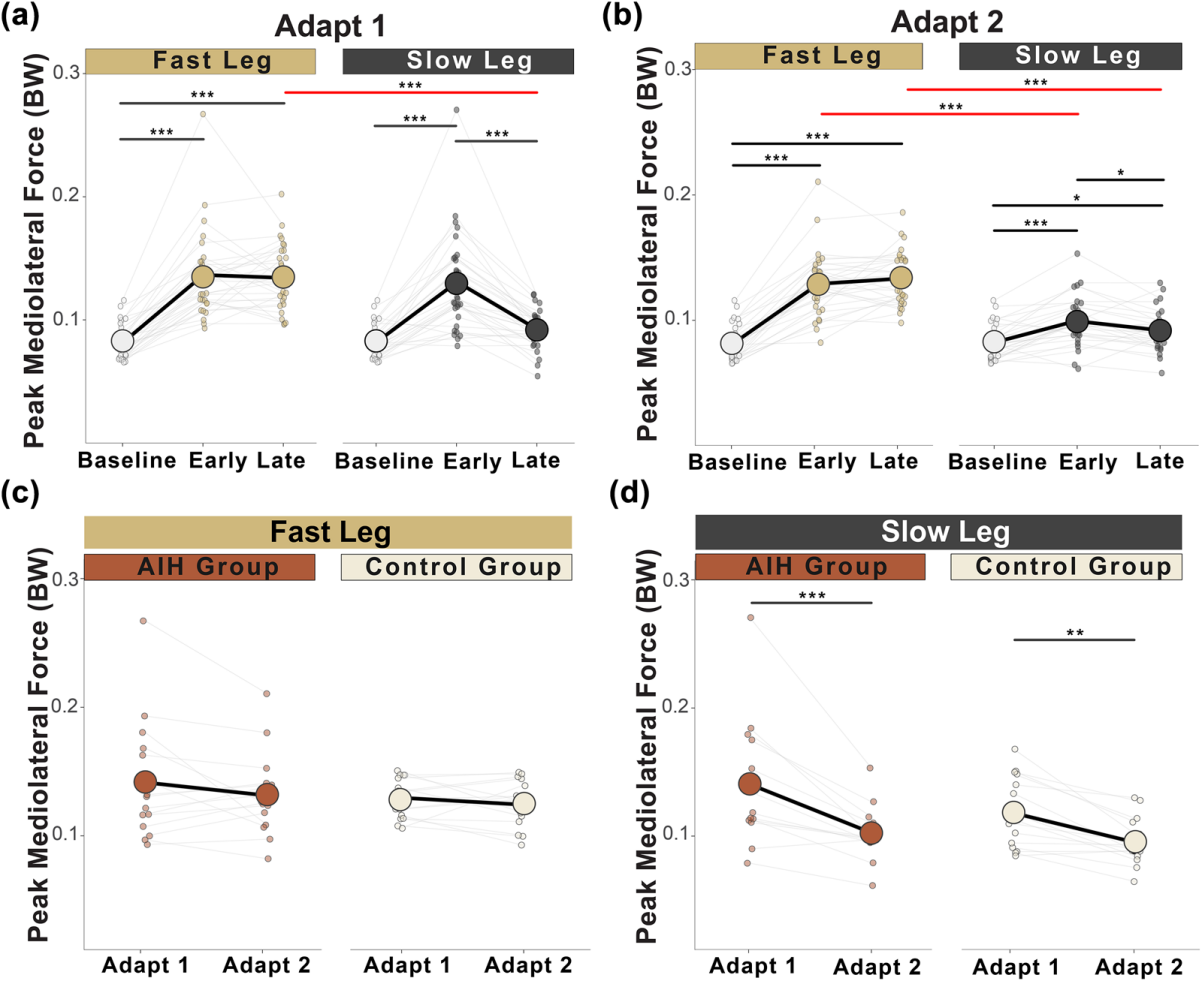
Peak mediolateral ground reaction forces during the braking phase. (a) In all participants (*n* = 30), peak ML GRF (in units of body weight) increased from baseline for both legs during early adaptation and decreased for the slow leg in late adaptation of adapt 1 in all participants (*n* = 30). (b) Between‐limb differences show higher peak ML GRF maintained by the fast leg during early and late adaptation of adapt 2 compared with the slow leg. (c, d) There were no early savings strategies observed for the fast leg (c), whilst the slow leg significantly decreased the magnitude of responses during adapt 2 for both groups (*n* = 15 AIH; *n* = 15 Control) (d). Circles represent mean peak ML GRF, and dots show the average peak ML GRF for each individual. *P < 0.05, **P < 0.01 and ***P < 0.001. The red line represents significant differences between limbs. Abbreviations: AIH, acute intermittent hypoxia; BW, body weight; GRF, ground reaction force; ML, mediolateral.

During adapt 1 and adapt 2, we observed main effects of leg speed, adaptation phase and interaction between leg speed and adaptation phase on peak ML GRF during the braking phase (Table 3). *Post hoc* analyses showed significant increases in force magnitudes for both legs during the braking phase from baseline to early adaptation, which decreased for only the slow leg during late adaptation (Figure 4a,b; Table 4).

#### 3.2.2 Motor adaptation during propulsive phase

**FIGURE 5:**
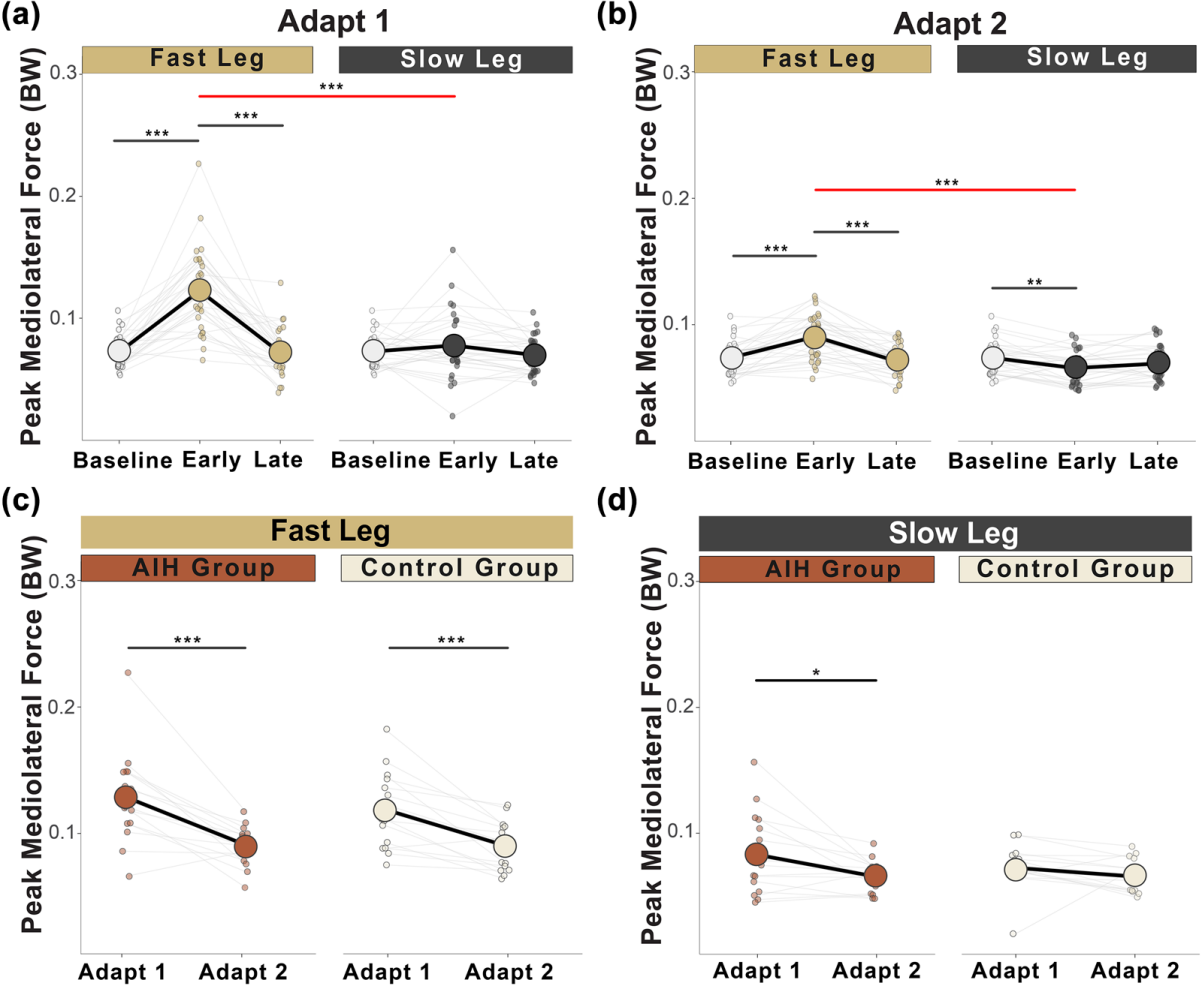
Peak mediolateral ground reaction forces during the propulsive phase. (a) In all participants (*n* = 30), only the fast leg increased peak ML GRF (in units of body weight) during early adaptation, which decreased in late adaptation of adapt 1 while the slow leg stayed at baseline. (b) During adapt 2, the fast leg showed a similar adaptation strategy to that in adapt 1, and the slow leg decreased peak ML GRF from baseline in early and late adaptation. (c) Both the AIH (*n* = 15) and Control (*n* = 15) groups showed early savings on the fast leg. (d) The AIH group further reduced force production on the slow leg. Circles represent mean peak ML GRF, and dots show the average peak ML GRF for each individual. *P < 0.05, **P < 0.01 and ***P < 0.001. The red line represents significant differences between limbs. Abbreviations: AIH, acute intermittent hypoxia; BW, body weight; GRF, ground reaction force; ML, mediolateral.

During adapt 1 and adapt 2, there were main effects of leg speed, adaptation phase and interaction of leg speed and adaptation phase on peak ML GRF during the propulsive phase (Table 3). Pairwise analysis showed significant increase in force magnitudes on the fast leg from baseline to early adaptation, which then decreased in late adapt 1 (Figure 5a) and adapt 2 (Figure 5b). The slow leg values remained near baseline during adapt 1 and decreased below baseline during early adapt 2 (Table 4).

### 3.3 Group differences during motor savings

#### 3.3.1 Enhanced step width reductions after AIH

To assess limb‐specific adaptations of step width in relationship to intervention, we analysed the fast and slow legs separately in the AIH and Control groups to evaluate motor savings strategies. For both the fast and slow legs, there was a main effect of adaptation phase, but no effect of group nor an interaction of phase and group (Table 3). Pairwise comparisons identified greater reductions in step width for both the fast and slow legs during early savings across repeated SBW tasks in the AIH group but not the Control group (Figure 3c,d; Table 4). We observed no late savings in either leg between phases, within groups, nor the interaction of these factors (Table S1).

In the Control group, no significant differences were observed between baseline and washout, indicating a return to baseline walking. In contrast, the AIH group adopted a narrower step width during washout (Table 5).

**TABLE 5:** Paired *t*‐tests between baseline and washout

			Mean (SD)				Student's paired *t*‐test	
			AIH		Control		AIH	Control
			Baseline	Washout	Baseline	Washout		
Step width, m		Right leg	0.153 (0.0156)	0.146 (0.0206)	0.168 (0.0261)	0.164 (0.0224)	*t*(14) = 2.30, *P =* 0.0373 *	*t*(14) = 0.929, *P =* 0.369
		Left leg	0.153 (0.0156)	0.146 (0.0204)	0.164 (0.0224)	0.164 (0.0224)	*t*(14) = 2.29, *P =* 0.0427 *	*t*(14) = 0.938, *P =* 0.364
Peak ML GRF, BW	Braking phase	Right leg	0.084 (0.014)	0.078 (0.018)	0.082 (0.016)	0.082 (0.015)	*t*(14) = 2.76, *P =* 0.0155 *	*t*(14) = 0.284, *P =* 0.781
		Left leg	0.084 (0.014)	0.076 (0.024)	0.082 (0.013)	0.086 (0.012)	*t*(14) = 1.74, *P =* 0.104	*t*(14) = −0.862, *P =* 0.403
	Propulsive phase	Right leg	0.075 (0.012)	0.073 (0.014)	0.077 (0.012)	0.077 (0.013)	*t*(14) = 1.45, *P =* 0.168	*t*(14) = 0.316, *P =* 0.757
		Left leg	0.070 (0.015)	0.070 (0.016)	0.072 (0.014)	0.074 (0.015)	*t*(14) = 0.157, *P =* 0.877	*t*(14) = −0.822, *P =* 0.425

*Note*: Mean (SD) and student's paired *t*‐test values for step width (in meters) and peak ML GRF (body weight) comparing baseline and washout for the right leg and left leg in the AIH (*n* = 15) and control (*n* = 15) groups. * *P* < 0.05, ** *P* < 0.01, *** *P* < 0.001. Abbreviations: AIH, acute intermittent hypoxia; BW, body weight; GRF, ground reaction force; ML, mediolateral.

#### 3.3.2 Savings of ML GRF adaptation strategies

To examine motor savings strategies during the braking phase between AIH and Control groups, peak ML GRF of the fast and slow legs were analysed independently across both SBW trials perturbations. During early and late savings, there were no main effects of adaptation phase, group, nor their interactions for the fast leg (Figure 4c). Conversely, for the slow leg we observed main effects of phase, but no effect of group nor interaction of group and phase during early savings (Table 3). Pairwise comparisons for the slow leg showed that both the AIH and Control groups demonstrated reductions of peak ML GRF during the braking phase (Figure 4d; Table 4).

In assessing motor savings between the AIH and Control groups during the propulsive phase, we observed a main effect of adaptation phase on peak ML GRF, but no group or interaction effects for both the fast and slow legs (Table 3). Pairwise analyses revealed that both groups exhibited early savings of peak ML GRF for the fast leg (Figure 5c), whereas only the AIH group demonstrated enhanced reductions on the slow leg (Figure 5d; Table 4).

No significant differences were found in peak ML GRF between baseline and washout during the braking phase in the Control group. In contrast, the AIH group exhibited lower force magnitudes on the right leg during washout, potentially as after‐effects of the prior adaptation. During the propulsive phase, no significant differences were observed in either group during the tied‐belt conditions, indicating that both legs resumed their baseline walking patterns (Table 5).

### 3.4 Metabolic adaptations

#### 3.4.1 Net metabolic power during motor adaptation and motor savings

**FIGURE 6:**
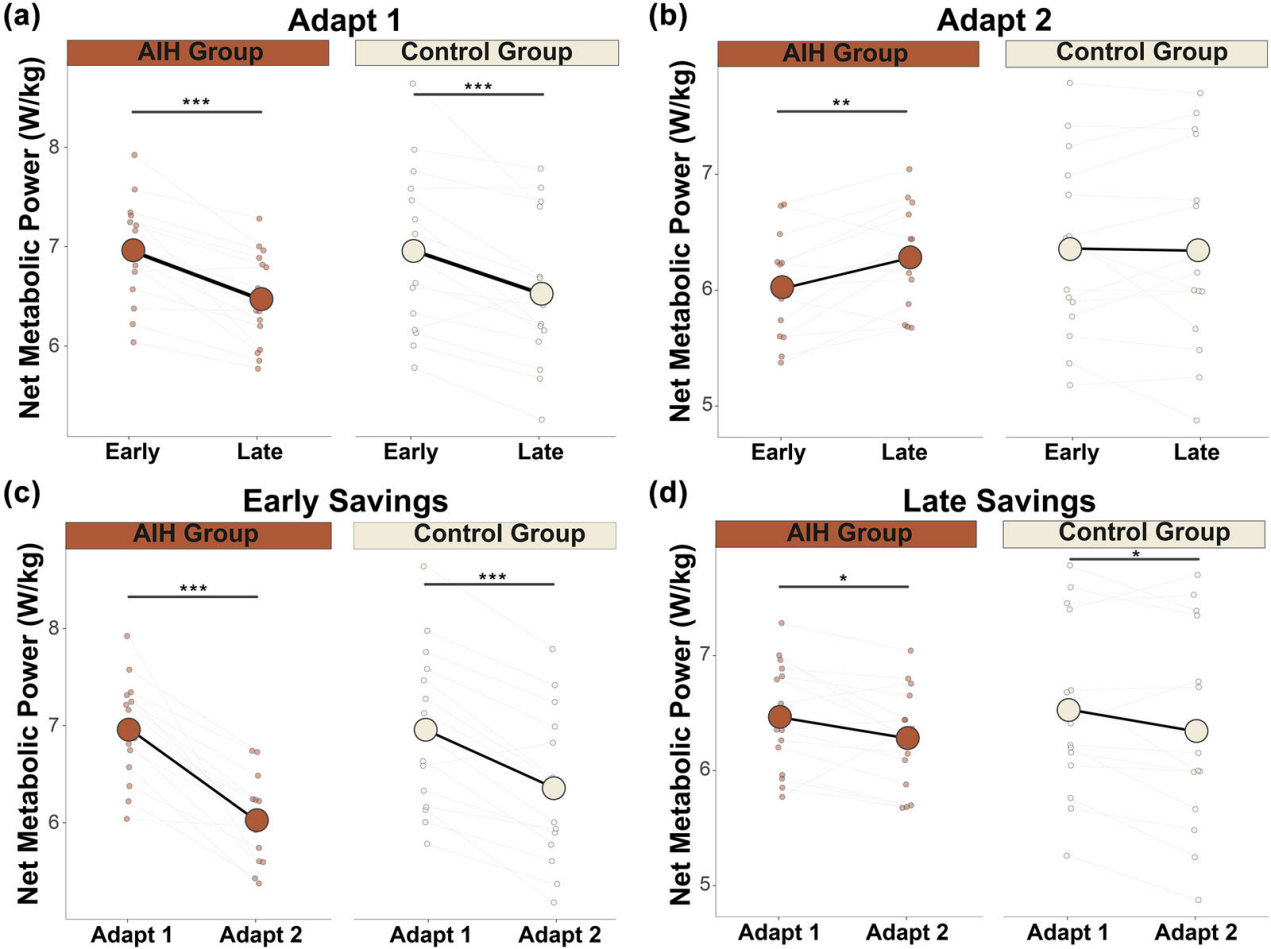
Net metabolic power. (a) Both groups (*n* = 15 AIH; *n* = 15 Control) decreased net metabolic power (in watts per kilogram) from early to late adaptation of adapt 1. (b) During adapt 2, the AIH group increased net metabolic power during late adaptation. (c, d) Both groups demonstrated early savings (c) and late savings (d) of net metabolic power. Circles represent mean net metabolic power, and dots show the average net metabolic power for each individual. *P < 0.05, **P < 0.01 and ***P < 0.001. Abbreviations: AIH, acute intermittent hypoxia.

During adapt 1, we observed main effects of adaptation phase on net metabolic power, but no group or interaction effects (Table 6). Student's paired *t*‐tests showed that net metabolic power decreased from early to late adaptation for both the AIH and Control groups (Figure 6a). During adapt 2, there were main effects of adaptation phase, and an interaction of group and phase. *Post hoc* comparisons revealed that the AIH group increased net metabolic power from early to late adaptation despite a lower mean net metabolic power compared with the Control group (Figure 6b; Table 6). LMMs performed across both adapt 1 and adapt 2 revealed main effects of phase and an interaction of group and phase during early savings and only a main effect of phase during late savings (Table 6). Pairwise analyses showed that both the AIH and Control groups demonstrated cost savings reflected as reduced net metabolic power during early and late savings (Figure 6c). The magnitude of reductions was significantly greater in the AIH group during early savings [*t*(24.3) = 2.15, *P* = 0.0420], whereas the difference between groups during late savings was not significant [*t*(27.9) = 0.028, *P* = 0.978].

**TABLE 6:** Net metabolic power analyses.

(a)		Net metabolic power (W/kg)				
		Adapt 1		Adapt 2		
		Early	Late	Early	Late	
Mean (SD)	AIH group	6.96 (0.516)	6.47 (0.475)	6.02 (0.430)	6.28 (0.427)	
	Control Group	6.96 (0.825)	6.53 (0.751)	6.36 (0.774)	6.34 (0.872)	
Mean difference (95% CI)		−0.002 (−0.517, 0.513)	0.229 (−0.411, 0.529)	0.228 (−0.138, 0.798)	0.251 (−0.452, 0.575)	
(b)		Linear mixed model main effects			Pairwise comparisons	
		Group	Phase	Group × phase	AIH group	Control group
Adapt 1		*F*(1,28) = 0.015, *P =* 0.903	*F*(1,28) = 42.5, *P =* 4.60 × 10^−7 ***^	*F*(1,28) = 0.184, *P =* 0.671	*t*(28) = 4.91, *P =* 3.53 × 10^−5 ***^	*t*(28) = 4.30, *P =* 1.84 × 10^−4 ***^
Adapt 2		*F*(1,28) = 0.708, *P =* 0.407	*F*(1,28) = 4.51, *P =* 0.0427 *	*F*(1,28) = 5.29, *P =* 0.0290 *	*t*(28) = −3.13, *P =* 0.00408 **	*t*(28) = 0.126, *P =* 0.901
Early savings		*F*(1,28) = 0.519, *P =* 0.477	*F*(1,28) = 98.6, *P =* 1.12 × 10^−10 ***^	*F*(1,28) = 4.61, *P =* 0.0406 *	*t*(28) = 8.54, *P =* 2.78 × 10^−9 ***^	*t*(28) = 5.50, *P =* 7.02 × 10^−6 ***^
Late savings		*F*(1,28) = 0.066, *P =* 0.799	*F*(1,28) = 10.7, *P =* 0.00278 **	*F*(1,28) = 7.63 × 10^−4^, *P =* 0.978	*t*(28) = 2.34, *P =* 0.0267 *	*t*(28) = 2.30, *P =* 0.0292 *

*Note*: (a) Mean (SD) and mean difference between groups (Control–AIH) with 95% confidence intervals of net metabolic power (in watts per kilogram) during early and late phases of adapt 1 and adapt 2. (b) Main effects and pairwise comparisons between phases (early adaptation vs. late adaptation) within adapt 1 and adapt 2 and across perturbations during early savings (early adapt 1 vs. early adapt 2) and late savings (late adapt 1 vs. late adapt 2) for the AIH (*n* = 15) and control (*n* = 15) groups. * *P* < 0.05, ** *P* < 0.01, *** *P* < 0.001.

Abbreviation: AIH, acute intermittent hypoxia.

#### 3.4.2 Associations between kinematic, kinetic and metabolic changes

**FIGURE 7:**
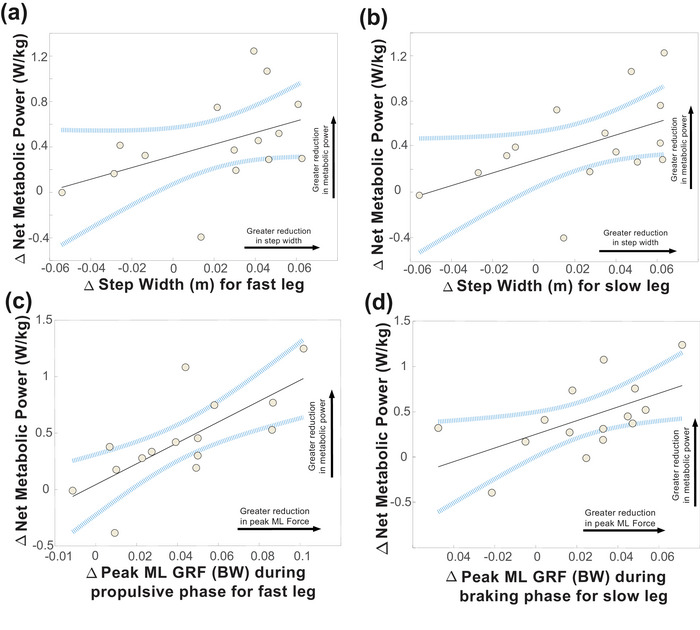
Metabolic regressions for fast and slow legs during adapt 1 in the Control group (*n* = 15). (a, b) Non‐significant relationship between reductions in step width (in metres) and reductions in net metabolic power (in watts per kilogram) was observed on the fast leg (a; Δnet metabolic power = 0.321 + 5.12 × Δstep width(fast leg), *R* ^2^ = 0.203, *P =* 0.092) and the slow leg (b; *y* = 0.293 + Δmetabolic power = 0.293 + 5.57 × Δstep width(slow leg), *R* ^2^ = 0.254, *P =* 0.055). (c, d) Larger decreases in peak ML force (in units of body weight) were significantly associated with greater reductions in net metabolic power during the propulsive phase on the fast leg (c; Δnet metabolic power = 0.0425 + 9.23 × Δpeak ML GRF(fast leg), *R* ^2^ = 0.540, *P =* 0.0018) and during the braking phase on the slow leg (d; Δnet metabolic power = 0.253 + 7.53 × Δpeak ML GRF(slow leg), *R* ^2^ = 0.328, *P =* 0.026). The area within blue lines represents 95% confidence, and grey points are data for individual participants. Abbreviations: AIH, acute intermittent hypoxia; BW, body weight; GRF, ground reaction force; ML, mediolateral.

We investigated associations between changes in net metabolic power and changes in kinematic and kinetic variables. There were non‐significant positive relationships between changes in net metabolic power and changes in step width during adapt 1 for both the fast (*P =* 0.0922) and slow leg (*P =* 0.0552) in the Control group (Figure 7a,b; Table S2). In contrast, significant correlations were observed between reductions in net metabolic power and decreases in peak ML GRF, notably during the propulsive phase for the fast leg (*P =* 0.00180; Figure 7c) and during the braking phase for the slow leg (*P =* 0.0256; Figure 7d) during adapt 1 in the Control group. There were no significant relationships within the AIH group (Table S2).

## 4 DISCUSSION

In this study, we examined adaptations in step width, peak ML GRF, and their association with changes in net metabolic power during split‐belt treadmill walking. We observe distinct coordination strategies in kinetic adaptations between the fast and slow legs during the initial perturbation exposure, in addition to positive associations between changes in peak ML GRF and reductions in net metabolic power. We also demonstrate motor savings of these interlimb adaptations during a subsequent perturbation. Notably, exposure to repetitive AIH further enhanced motor savings of adaptive frontal plane mechanics.

### 4.1 Step width reductions during motor adaptation

To our knowledge, this is the first study that demonstrates progressive reductions in step width across both initial exposure and re‐exposure to SBW. Congruent with our hypothesis, participants used an initial compensatory widening of gait to preserve stability under the newly altered belt speeds (McAndrew Young et al., 2012). Prior studies likewise observed initial increases in step width (Fettrow et al., 2021), in addition to increases in the ML margin of stability during early adaptation (Brinkerhoff et al., 2024; Buurke et al., 2018), followed by reductions in step width throughout motor adaptation. However, the present study extends these findings by demonstrating persistent reductions upon re‐exposure, reflecting retrieval of a previously retained ML control strategy.

### 4.2 Distinct interlimb adaptation patterns in peak ML GRF during motor adaptation

The fast and slow legs differentially adapted peak ML GRF during the braking phase of both SBW trials. The braking phase of gait, occurring immediately after heel‐strike in early stance, is crucial for re‐stabilization and might explain why the fast leg maintained higher peak ML forces during this phase (Rawal & Singer, 2021). Likewise, our group previously noted larger braking forces on the fast leg in the sagittal plane during SBW (Bogard et al., 2023). In contrast, the leg on the slow belt adopted a different strategy, by decreasing peak force magnitudes as adaptation progressed. These findings emphasize the unique role of each leg in maintaining lateral control of stability during SBW adaptation. The distinct interlimb adaptations observed might be attributed, in part, to independent neural control mechanisms for each limb, which are modulated by limb‐specific sensory feedback (Choi & Bastian, 2007). Moreover, evidence that intralimb adaptations occur at a faster time scale than interlimb coordination (Sato & Choi, 2022) might explain the persistence of asymmetrical gait patterns. Contrary to our findings, Roper et al. (2017) observed no interlimb differences in ML GRF impulses during the braking phase. This discrepancy might arise from the limitation that time‐integrated force values might obscure transient force fluctuations that are better captured by peak magnitudes, especially in dynamically adaptive environments (Deffeyes & Peters, 2021).

During the propulsive phase, adaptation is asymmetric such that peak ML GRF of the fast leg is modulated across early to late adaptation, while the slow leg remains near baseline during adapt 1 and adapt 2. These distinct interlimb responses in ML GRF correspond to the respective belt speed for each leg, resembling the force magnitudes observed during tied‐belt walking at matched speeds (Roper et al., 2017). Consistent with prior reports, the magnitude of ML kinetic adaptations observed in the present study was relatively small compared with changes in AP force production in response to split‐belt perturbations. Specifically, ML forces have been shown to increase by ∼0.05–0.1 × BW (Roper et al., 2017), whereas adaptations in peak AP propulsive forces are greater in magnitude, increasing by ~0.1–0.2 × BW (Bogard et al., 2023; Sombric et al., 2019). These differences suggest that ML control contributes less to the overall energetic demand than sagittal‐plane force generation, as AP propulsion is directly associated with centre‐of‐mass work and positive mechanical power production that underlie metabolic cost (Donelan et al., 2002; Grabowski et al., 2005).

### 4.3 Correlations between kinematics, kinetics and net metabolic power

Although bilateral narrowing of step widths was not correlated with reductions in net metabolic power, other studies have reported reductions in metabolic cost when frontal plane strategies are optimized to walk at preferred step width (Donelan et al., 2001) and when participants exploited frontal plane passive dynamics (Fettrow et al., 2021). In the sagittal plane, reductions in net metabolic power parallel increases in step time asymmetry and decreases in step length asymmetry during motor adaptation and savings (Bogard et al., 2023; Sánchez et al., 2019). These findings suggest that metabolic changes during SBW are driven by distinct yet complementary kinematic adaptations across both planes, in addition to limb‐specific kinetics that are likely to be interdependent (Buurke & den Otter, 2021; Buurke et al., 2020). Although we do not observe significant associations in step width, peak ML GRF during propulsion was significantly correlated with net metabolic power, indicating that ML force control during push‐off plays a crucial role in regulating metabolic cost during SBW adaptation. One consideration is that step width adjustments respond more quickly to perturbations to maintain stability and are driven primarily by sensory feedback, whereas adaptations in force occur gradually, over longer time scales, reflecting feedforward control (Mawase et al., 2013). However, we restrict our analyses to steady‐state metabolic data owing to time delays in the measurement system, a limitation not unique to our protocol (Arellano & Kram, 2011; Gottschall & Kram, 2003; Grabowski et al., 2005). By omitting the first 1 min of each walking condition, it precludes an estimation of the early cost that is exclusively associated with initial, rapid adjustments (Bogard et al., 2023; Finley et al., 2013). Nevertheless, the present findings do not rule out the possibility that step width adaptations are reflected in changes in metabolic power, because kinematic adjustments have been shown to contribute to overall cost during walking (Donelan et al., 2004; Wu et al., 2020). Although these associations do not demonstrate causality, the significant relationship with ML force indicates that adaptations in frontal plane kinetics are a greater driver of reductions in energetics than ML kinematic changes.

The observed reductions in metabolic cost are likely to reflect the contributions of changes in mechanical adaptations in both sagittal and frontal planes. Thus, a limitation of the present study is that the individual contributions of each adaptation to the overall reduction in net metabolic power cannot be isolated. Indeed, previous studies have demonstrated that both active control of frontal plane mechanics (Donelan et al., 2004) and the modulation of propulsive force in the sagittal plane have considerable influence on the overall metabolic expenditure during walking energetics (Grabowski et al., 2005; Pieper et al., 2021). Regulation of frontal plane stability is key in driving initial adaptation, because recent studies show that ML adjustments adapt at a faster time scale than in the sagittal plane (Brinkerhoff et al., 2024). Furthermore, it has been proposed that walking with more positive step length asymmetry resulted in increased lateral whole‐body momentum, indicating that improvements in sagittal plane stability might come at the negative expense of frontal plane stability (Cornwell et al., 2024), a pattern also observed in the post‐stroke of individual (Buurke et al., 2020). Together with our findings, these observations emphasize the importance of examining adaptations across both planes to gain a comprehensive understanding of how their interactions influence energetics.

Both limb‐ and phase‐specific adaptive strategies observed during the braking and propulsive phases indicate distinct mechanical roles that contribute to both stability and energetic efficiency. Based on our observations, ML forces generated during the propulsive phase contribute to metabolic adaptation, underscoring the potential role of optimizing push‐off mechanics in reducing energy expenditure. For example, application of external lateral stabilization reduced energetic costs by minimizing excessive ML movement (Dean et al., 2007), and external horizontally applied forces similarly lowered metabolic rate by offsetting the high energy cost of generating propulsive forces during walking (Gottschall & Kram, 2003). Interestingly, increasing propulsive demand through inclined SBW appeared to improve the magnitude of motor adaptation in the sagittal plane in participants with stroke, as evidenced by reductions in step length asymmetry (Sombric & Torres‐Oviedo, 2020). This suggests that the kinetic demands of propulsion not only impact the energetic cost but also shape the magnitude of motor adaptation. Altogether, these parallel observations suggest an interplay between AP adjustments and peak ML force patterns, supporting the interpretation that more efficient coordination strategies across planes collectively contributed to reductions in metabolic cost.

### 4.4 Enhanced step width reductions following AIH

The AIH group uniquely demonstrated greater reduction of step width during early savings in both the fast and slow legs, suggesting that low‐oxygen intervention primes the nervous system to facilitate enhanced retention of previously learned strategies in the frontal plane. These findings corroborate our previous observations that repetitive AIH improves motor savings of spatiotemporal asymmetry in the sagittal plane (Bogard et al., 2023). We observed no late savings in either group, reflecting a consolidation of adaptation strategies at an earlier time scale regardless of intervention. Frontal plane adjustments rely on multiple neural control processes that also shape sagittal plane mechanics and adapt at different time scales (Brinkerhoff et al., 2024). Given evidence of AIH‐induced neuroplasticity (Christiansen et al., 2018; Finn et al., 2022) and improved motor performance (Bogard et al., 2024, 2025), enhanced activation of the neural processes involved in frontal plane control might contribute to the observed improvements in early ML adaptations. As such, effects attributable to AIH might be captured more clearly during early phases of motor learning, whereas late adaptation seems to be less influenced by neuromodulatory mechanisms. This interpretation is congruent with prior work demonstrating that visuomotor adaptations are more prominent at the onset compared with the end of optic flow perturbations, suggesting that early phase adaptations are particularly responsive to perturbations (Thompson & Franz, 2017).

Interestingly, the AIH group demonstrated reduced step widths during washout, whereas control participants showed a return to baseline values. Persistence of this narrowed step‐width strategy suggests after‐effects of prior SBW adaptation. Such after‐effects have been proposed to reflect the ability to adapt new gait patterns. For instance, older adults exhibited fewer after‐effects of step length symmetry upon returning to tied‐belt walking, indicating a reduced capability for adaptation (Bruijn et al., 2012). Furthermore, older individuals experienced reduced fall rates after a virtual reality gait training intervention (Mirelman et al., 2016). Together, these findings suggest that AIH intervention might facilitate locomotor adaptation.

### 4.5 Motor savings: Reduced peak ML GRF

Upon re‐exposure to the SBW task, individuals in both groups exhibited early savings during the braking phase, reflected by reduced forces on the slow leg. In contrast, early savings during the propulsive phase were uniquely enhanced on the slow leg following AIH. These distinct adaptive responses demonstrate leg‐ and phase‐specific contributions to asymmetric gait adaptation, wherein the braking phase supports restabilization following foot contact (Rawal & Singer, 2021), whereas the propulsive phase allows for interlimb force modulation to support forward progression. These findings demonstrate that AIH enhances early savings of interlimb control strategies, which are more pronounced during propulsion. Consistent with these results, AIH similarly induces greater reductions of AP propulsive force asymmetry (Bogard et al., 2023). Although AP asymmetry was previously shown ultimately to reduce during SBW (Bogard et al., 2023), interlimb differences in frontal plane kinetics persisted, suggesting that ML control strategies might be influenced by unique neural and mechanical demands (Cornwell et al., 2024). Thus, plane‐specific adaptations might also reflect distinct control priorities, in which sagittal plane adjustments prioritize symmetry for forward propulsion, whereas frontal plane adaptations maintain asymmetrical mechanics to preserve stability.

We observed no significant motor savings during the late adaptation phases, indicating that the coordination strategies of participants had converged to a preferred walking pattern. Consistent with this view, other SBW studies have also demonstrated plateaued adaptations during late adaptation (Bogard et al., 2023; Buurke et al., 2022; Leech et al., 2018).

### 4.6 Reductions in energetic demand

We observed that both groups concurrently reduced net metabolic power as they adapted their frontal plane mechanics, supporting the notion that adaptive control of dynamic balance is related to the reduction in energy expenditure (Buurke et al., 2018; Donelan et al., 2001; Finley et al., 2013; Selinger et al., 2015). Importantly, the reduction in energetic cost during early savings was significantly greater following AIH, further indicating that priming the nervous system enhances retrieval of motor strategies. Although we observed increases in net metabolic power in the AIH group during late learning in adapt 2, they still used ∼0.3 W/kg less than the Control group during early learning, probably pointing to more economical gait strategies facilitated by motor savings and improved frontal plane control. Consistent with prior studies, re‐exposure to perturbation leads to more energy‐efficient gait patterns, such as reduced step length asymmetry (Buurke et al., 2022; Finley et al., 2013; Roemmich & Bastian, 2015), optimization of step frequency (Selinger et al., 2015), decreased AP force asymmetry (Bogard et al., 2023) and reductions in positive mechanical work (Sánchez et al., 2019). Propulsive forces, in particular, have been shown to drive motor adaptation during split‐belt walking in both healthy individuals (Sombric et al., 2019) and neurologically impaired populations (Hagen et al., 2024; Sombric & Torres‐Oviedo, 2020). Additionally, reductions in propulsive demand are associated with decreased metabolic cost during walking (Grabowski et al., 2005). Consistent with these findings, we observed pronounced motor savings during the propulsive phase following AIH, suggesting that adaptation during this phase might be targeted preferentially to minimize metabolic cost.

Notably, the findings of the present study, which used a 2:1 speed ratio, contrast with our previous observations using a 1.5:1 speed ratio, in which we noted continual reductions in net metabolic power during the subsequent SBW (Bogard et al., 2023). Given previous work showing that savings of biomechanical adaptations are improved with exposure to larger perturbations that use greater split‐belt speed ratios (Leech et al., 2018), we speculate that energetic savings might reach a plateau as split‐belt speed ratios increase.

### 4.7 Improved adaptations following AIH

We demonstrated enhanced motor savings of both kinematic and kinetic adaptations following repetitive AIH. Notably, the AIH group exhibited greater reductions in step width for both legs, in addition to greater reductions in peak ML GRF during the propulsive phase for the slow leg. These results are consistent with our previous sagittal plane findings (Bogard et al., 2023), suggesting that AIH also engages neural processes that influence ML control. However, the specific neural mechanisms underlying AIH‐induced improvements in motor performance and motor learning remain unclear. Evidence from spinally injured rats and human studies suggest that AIH increases brain derived neurotrophic factor (BDNF)‐dependent synaptic plasticity (Lovett‐Barr et al., 2012) and facilitates descending neural excitability (Bogard et al., 2023, 2025; Christiansen et al., 2018). Increases in volitional drive to the soleus muscle have been reported following AIH, indicating that this motor pathway is responsive to neuromodulation (Bogard et al., 2024). Given converging evidence that soleus activation contributes substantially to forward propulsion during walking (Ellis et al., 2014), AIH‐induced increases in descending neural excitability may drive more pronounced adaptations during the propulsive phase. Collectively, these findings support the plausible interpretation that AIH‐induced increases in BDNF promote sensorimotor adaptation (Bogard et al., 2025), which might contribute to the enhanced motor savings observed in the present study. These effects parallel similar BDNF‐dependent mechanisms that might underlie improvements across different mechanisms of motor learning, including visuomotor adaptation and use‐dependent plasticity (Fritsch et al., 2010; Helm et al., 2016; Joundi et al., 2012; Mang et al., 2014).

Previous research suggests that the nervous system facilitates postural adjustments by sending commands to anticipate and react to postural threats (Cesari et al., 2022). Motor savings in step width and peak ML GRF reductions might indicate a shift from generalized anticipatory strategies to more task‐specific reactive control to preserve stability following a perturbation (Ahuja & Franz, 2022; Ogawa et al., 2014). These observations further suggest that with repeated exposure to the perturbation, the nervous system refines frontal plane control mechanisms to rely more on reflexive adjustments. Although we did not measure variability between consecutive steps, quantifying persistent increases in step variability throughout adapt 2 might further clarify shifts towards task‐specific reactive control that accommodates flexible foot placement strategies (Ahuja & Franz, 2022). Although the present dataset cannot parse the underlying neural mechanisms, it remains plausible that AIH‐induced synaptic plasticity contributes to enhanced adaptive control, leading to more effective control of stability during walking in environments with external perturbations.

AIH has significant potential for integration into the design of rehabilitation training protocols aimed at improving walking stability and reducing fall risks. Indeed, both long‐term and progressive training protocols have been demonstrated previously to induce improvements in motor learning (Christiansen et al., 2020), lateral balance control (Sawers et al., 2013) and enhanced walking performance following repetitive AIH (Hayes et al., 2014; Tan et al., 2021). Moreover, prolonged exposure to optical flow perturbations has the potential to be used as a training method to improve corrective motor adjustments while walking in older adults (Richards et al., 2019). Future studies might implement targeted practice protocols combined with AIH to improve dynamic balance in persons with neurological deficits, such as spinal cord injury (Navarrete‐Opazo et al., 2017), in addition to healthy older adults. Our findings underscore the coupling between adaptive ML control and walking energetics, which might further inform the detection of balance decline and the tailoring of patient‐specific rehabilitation strategies to improve postural control (Selgrade et al., 2020).

## 5 CONCLUSION

This study investigated changes in frontal plane kinematics and kinetics during an initial exposure and re‐exposure to SBW. We observed bilateral increases in step width, which returned to baseline as individuals adapted to SBW. Conversely, asymmetries in peak ML forces were maintained throughout adaptation during both the braking and propulsive phases of gait and were positively correlated with reductions in net metabolic power. These results reveal distinct ML control strategies that regulate stability and reduce energetic demand. Notably, AIH exposure augmented retention of these adaptive coordination strategies upon re‐exposure to SBW. These findings support the integration of AIH as a neuromodulatory adjuvant for rehabilitation interventions that target clinical populations with impaired balance control.

## AUTHOR CONTRIBUTIONS

This study was performed in the NeuroRecovery Lab at CU Boulder. Andrew Q. Tan and Alysha T. Bogard designed the study protocol. Alysha T. Bogard, Aviva K. Pollet and Norah M. Nyangau conducted the experiments. Alysha T. Bogard and Norah M. Nyangau analysed the data. Norah M. Nyangau drafted the original manuscript. All authors contributed to the interpretation of the results and revision of the manuscript. All authors approved the final version of the manuscript and agree agree to be accountable for all aspects of the work in ensuring that questions related to the accuracy or integrity of any part of the work are appropriately investigated and resolved. All persons designated as authors qualify for authorship, and all those who qualify for authorship are listed.

## CONFLICT OF INTEREST

None declared.

## Supporting information



Supporting Information

## Data Availability

The authors confirm that the data supporting the findings of this study are fully available and presented in the Supporting Information of the manuscript. Correspondence and requests for materials should be addressed to A.Q.T.
