# Supplementary material for: Adaptive mediolateral control during split‐belt walking: Energetics of interlimb coordination and enhanced savings following acute intermittent hypoxia
Source: Exp Physiol. 2026 Mar 21;111(5):2645–64. doi: 10.1113/EP093291 (PMC13131107; doi:10.1113/EP093291)
Supplement: Supplementary file 1 — Supporting Information [file EPH-111-2645-s001.docx]

**SUPPLEMENTARY INFORMATION**

|  | | **Late Savings Main Effects** | | | | | |
| --- | --- | --- | --- | --- | --- | --- | --- |
|  | | **Fast Leg** | | | **Slow Leg** | | |
|  | | **Group** | **Phase** | **Group x Phase** | **Group** | **Phase** | **Group x Phase** |
| **Step Width (m)** | | *F*(1,28) = 4.08,  *p =* 0.0530 | *F*(1,28) = 0.508,  *p =* 0.482 | *F*(1,28) = 2.52,  *p =* 0.123 | *F*(1,28) = 3.82,  *p =* 0.0606 | *F*(1,28) = 0.722,  *p =* 0.403 | *F*(1,28) = 2.57,  *p =* 0.120 |
| **Peak ML GRF (BW)** | **Braking Phase** | *F*(1,28) = 0.002  *p =* 0.965 | *F*(1,28) = 0.099,  *p =* 0.756 | *F*(1,28) = 0.200,  *p =* 0.658 | *F*(1,28) = 0.972,  *p =* 0.332 | *F*(1,28) = 0.003, *p =* 0.955 | *F*(1,28) = 0.0004,  *p =* 0.983 |
|  | **Propulsive Phase** | *F*(1,28) = 0.723,  *p =* 0.402 | *F*(1,28) = 0.022,  *p =* 0.884 | *F*(1,28) = 1.46,  *p =* 0.238 | *F*(1,28) = 0.021,  *p =* 0.885 | *F*(1,28) = 0.003 *p =* 0.953 | *F*(1,28) = 0.008  *p =* 0.927 |
|  | | | | | | | |

**Supplementary Table S1. Late savings analyses.** Linear Mixed Model of late savings (late adaptation during adapt 1 vs late adaptation during adapt 2) main effects of group and condition on step width (m) and peak ML GRF (body weight, BW) during the braking and propulsive phases for the fast and slow legs for the AIH (*n* = 15) and Control (*n* = 15) groups.

|  |  | 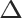 **Net Metabolic Power (W/kg)** | | | | | | | |
| --- | --- | --- | --- | --- | --- | --- | --- | --- | --- |
|  |  | **AIH Group** | | | | **Control group** | | | |
|  |  | **Adapt 1** | | **Adapt 2** | | **Adapt 1** | | **Adapt 2** | |
|  |  | **R^2^** | ***P*-value** | **R^2^** | ***p*-value** | **R^2^** | ***P*-value** | **R^2^** | ***p*-value** |
| 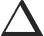 **Step  Width (m)** | **Fast**  **Leg** | 8.94 x 10^-2^ | 0.279 | 4.79 x 10^-2^ | 0.433 | 0.203 | 0.0922 | 5.69 x 10^-2^ | 0.392 |
|  | **Slow Leg** | 8.94 x 10^-2^ | 0.279 | 0.107 | 0.234 | 0.254 | 0.0552 | 7.17 x 10^-2^ | 0.335 |
| 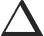 **Peak ML GRF during Braking Phase (BW)** | **Fast  Leg** | 2.38 x 10^-3^ | 0.863 | 6.12 x 10^-2^ | 0.374 | 2.39 x 10^-2^ | 0.582 | 0.183 | 0.111 |
|  | **Slow Leg** | 3.99 x 10^-3^ | 0.823 | 0.118 | 0.210 | 0.328 | **0.0256 *** | 6.30 x 10^-2^ | 0.367 |
| 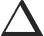 **Peak ML GRF during Propulsive Phase (BW)** | **Fast  Leg** | 2.75 x 10^-2^ | 0.555 | 7.18 x 10^-7^ | 0.998 | 0.728 | **0.001 **** | 0.118 | 0.211 |
|  | **Slow Leg** | 5.58 x 10^-2^ | 0.396 | 1.97 x 10^-2^ | 0.618 | 0.129 | 0.188 | 2.06 x 10^-2^ | 0.610 |

**Supplementary Table S2. Linear regression analyses.** Correlations between changes in net metabolic power and changes in step width (m) and peak ML GRF (body weight, BW) during the braking and propulsive phases were assessed for the fast and slow legs by calculating ordinary R^2^ and *p-*values in both the AIH (*n* = 15) and Control (*n* = 15) groups. * *P* < 0.05, ** *P* < 0.01, *** *P* < 0.001.
